# Only low-arousal emotions matter: grade-dependent mediation of academic emotions between psychological resilience and learning burnout in middle school

**DOI:** 10.3389/fpsyg.2026.1891219

**Published:** 2026-08-07

**Authors:** Jian-jun Zhong, Yanli He, Kuan Li

**Affiliations:** 1 School of Vocational Education, Tianjin University of Technology and Education, Tianjin, China; 2 Psychological Counseling Center, Lanzhou Resources and Environment Voc-Tech University, Lanzhou, Gansu, China; 3 School of Humanities, Inner Mongolia Polytechnic University, Inner Mongolia Autonomous Region, Hohhot, China

**Keywords:** learning burnout, negative academic emotions, positive academic emotions, primary middle school students, psychological resilience

## Abstract

**Background:**

Psychological resilience is known to reduce learning burnout, but it remains unclear whether low- versus high-arousal academic emotions mediate this relationship and whether such effects vary across grades.

**Objective:**

This study examined the mediating roles of academic emotions and tested grade differences.

**Method:**

A cluster sample of 739 middle school students (grades 7–9) from urban and rural schools completed three validated scales assessing psychological resilience, learning burnout, and academic emotions (positive/negative × high/low arousal). Structural equation modeling with bias-corrected bootstrapping was used to test mediation and grade differences.

**Results:**

Resilience significantly predicted learning burnout. Only low-arousal academic emotions (both positive and negative) served as significant partial mediators, whereas high-arousal emotions did not. Notably, positive low-arousal emotions (e.g., calmness, contentment) mediated the resilience–burnout relationship exclusively in grade 9, with no such effect in grades 7 or 8. Negative low-arousal emotions (e.g., boredom) mediated consistently across all grades.

**Conclusion:**

Resilience reduces burnout through low-arousal emotions. Negative low-arousal emotions are a consistent mediator throughout middle school, but positive low-arousal emotions emerge as protective only in grade 9, highlighting the need for grade-specific interventions.

## 1 Introduction

Despite the initiatives undertaken by China’s Ministry of Education to alleviate the academic workload since 2018, middle school students’ academic pressure continued to rise through 2020 (Wang and Yang, 2022). Elevated stress levels can increase negative emotions and undermine self-functions, which ultimately results in learning burnout (Martínez et al., 2021; Wang et al., 2021; Huang et al., 2023) which in turn may cause cognitive impairments and brain alteration (Bayes et al., 2021). Recent evidence further suggests that emotions such as enjoyment and anxiety are closely associated with learners’ self-efficacy and engagement (Li et al., 2025; Sun et al., 2026), underscoring the importance of understanding emotional mechanisms in diverse academic settings. Importantly, academic stress and its impact vary across grades (Liu, 2016). A developmental aspect that remains critically underexplored in the context of burnout.

To address this dilemma, the Demand-Resources Model (Bakker and Demerouti, 2014) suggests that personal resources such as psychological resilience which is capacity to cope with adversity (Olsson et al., 2003) can buffer the negative effects of demands burnout. However, the mechanisms through which resilience operates remain insufficiently understood. Academic emotions, which are closely linked to learning stress and self-evaluation (Yan and Yu, 2007; Jayanthi et al., 2015), serve as key mediators. Specifically, the type of emotion (positive vs. negative, high- vs. low-arousal) could determine the nature of the mediating effect, and these pathways are likely to be moderated by grade level due to developmental shifts in emotion regulation.

This study aims to bridge these gaps by examining: (a) whether low- versus high-arousal academic emotions mediate the relationship between psychological resilience and learning burnout, and (b) whether these mediating mechanisms differ across middle school grades. By doing so, we seek to provide empirical evidence for the development of grade-specific, emotion-focused interventions to combat learning burnout.

## 2 Literature review and hypotheses development

### 2.1 Learning burnout and its relationships with psychological resilience

Burnout is a pathological condition characterized by emotional and physical exhaustion resulting from prolonged work-related stress (Freudenberger, 1974). Research has shown that students similarly experience burnout due to academic challenges, competition, and interpersonal dynamics, leading to symptoms such as fatigue and cynicism. The construct of learning burnout is conceptualized as a three-dimensional framework that includes a perceived lack of control over the learning process, depersonalization and a reduced sense of achievement (Schaufeli et al., 2002; Erturguta and Soysekerci, 2010).

Within the framework of the stress Demand Resources Model, learning burnout is influenced not only by academic stressors but also by psychological capital and how individuals approach academic tasks. Direct factors such as academic workload, task difficulty, and social support are linked to burnout (Salanova et al., 2011; Yu et al., 2016), while indirect factors include perfectionism, success expectations, and academic emotions (Yang and Chen, 2016; Sorkkila et al., 2017; Wang and Zheng, 2023). Protective factors—such as self-efficacy, self-esteem, coping strategies, and emotional regulation also play a critical role.

Psychological resilience involves the capacity to effectively utilize internal and external resources to cope with adversity, maintain self-efficacy, and adapt under challenging conditions (Block and Block, 1980). It not only encompass the evaluation of situations, processing of inner experiences, generation of meaning, and the establishment of beliefs that guide actions (Rutter, 1985; Luthar et al., 2000; Güldener, 2018), but also emotional regulation, self-acceptance, social support, frustration management, and individual grit (Anderson and Priebe, 2021; Tang et al., 2021). Psychological resilience serves as a buffer against the negative effects of stress, frustration, and psychological trauma and a facilitator of personal growth, it constitutes a vital component of resources within the Demand Resources Model (Bakker and Demerouti, 2014).

Previous studies have demonstrated that psychological resilience is a significant negative predictor of learning burnout (Chen, 2016; Selim, 2023), with its dimensions exhibiting a significant negative correlation with learning burnout and its associated dimensions (Chen, 2016; Liu et al., 2018). The roles of psychological resilience in mitigating learning burnout are likely attributed to its capacity to continuously foster adaptive emotional regulation, sustain self-esteem and self-efficacy, and help students navigate academic difficulties. Thus, psychological resilience not only reduces the risk of burnout but also supports continued academic engagement and wellbeing.

### 2.2 The mediation of academic emotions between psychological resilience and learning burnout

Academic emotions are tied to specific educational contexts (e.g., learning tasks, achievement goals) and encompass both positive and negative feelings related to learning, teaching, and achievement (Linnenbrink and Pintrich, 2002; Pekerun et al., 2002). These emotions arise from sources such as enjoyment, boredom, hope, helplessness, pride, or shame. Once activated, these emotions significantly influence self-regulated learning, shaping comprehension, engagement, and behavioral investment (Pekrun, 2006).

Studies show that positive academic emotions mitigate learning burnout, while negative emotions exacerbate it (Gao, 2014; Wang and Zheng, 2023). Specifically, positive high-arousal and negative low-arousal emotions play opposing roles (Ruoyi Qu et al., 2022). Specific emotions, such as hatred, have been identified as mediators in the progression from emotional exhaustion to cynicism (Lee et al., 2020) The Broaden-and-Build Theory suggests that positive emotions enhance learning strategies and motivation (King and Areepattamannil, 2014). while negative emotions impair cognitive evaluations and increase burnout (Deng et al., 2022; Zyberaj, 2022). Extending these findings to the context of resilience, psychological resilience not only directly reduces learning burnout but also exerts an indirect influence through the mediation of positive academic emotions (Gao et al., 2018); similarly, it alleviates the detrimental effects of negative academic emotions (Ye et al., 2017; Liu et al., 2019). In summary, academic emotions—shaped by perceptions of control, stress, and self-evaluation—are closely tied to the development of burnout, and their impact is well established at the valence level. These findings, however, are largely confined to the valence dimension, leaving the role of emotional arousal largely unaddressed.

While valence has been extensively studied, focusing solely on it is insufficient to understand the chronic process of learning burnout. Based on the circumplex model of affect (Russell, 1980) and the Motivated Attention framework (Lang et al., 1998), the arousal dimension—ranging from activating (high-arousal) to deactivating (low-arousal)—offers a more nuanced lens for examining the emotion–burnout link. In the context of persistent academic demands, low-arousal emotions are theoretically more consequential as mediators than their high-arousal counterparts for three interconnected reasons. First, high-arousal emotions (e.g., anxiety, excitement) are typically elicited by acute events and decay rapidly, whereas low-arousal emotions (e.g., calmness, boredom) are more enduring, sustained by chronic features of the academic environment, and thus better aligned with the prolonged development of burnout (Verduyn et al., 2009; Kuppens et al., 2010). Second, chronic academic stress manifests as sustained low-grade HPA axis activation, closely associated with deactivating low-arousal affect, rather than acute high-arousal spikes (Lupien et al., 2009; Bayes et al., 2021). Third, low-arousal positive emotions facilitate reflective problem-solving, while low-arousal negative emotions deplete resources through passive rumination; high-arousal states, by contrast, may narrow attentional scope or paradoxically mobilize effort (Gable and Harmon-Jones, 2010). Thus, the arousal dimension accounts for temporal stability, neurobiological correlates, and cognitive affordances, offering superior explanatory power for understanding how resilience-induced emotional states translate into protective or destructive trajectories over time. However, whether both high- and low-arousal emotions—regardless of valence—serve as mediators between resilience and burnout remains underexplored. Further investigation is needed to examine these differential effects across arousal levels.

### 2.3 The differences of academic contexts among grades

Academic demands evolve significantly across the 3 years of middle school. The first-year students face challenges related to transitioning from primary education, including adapting to a new learning environment, mastering multidisciplinary content, and acquiring new learning strategies. By the second year, students have generally adjusted to the school setting but are required to manage a broader range of subjects. In the third year, academic pressures intensify due to preparation for high school entrance examinations and an increased volume of learning content.

Research on academic stress among middle school students indicates that levels vary by grade. Students in grades one and three report higher stress across multiple dimensions including task demands, frustration, competition, expectations, and self-development pressure compared to their grade two counterparts (Liu, 2016). Moreover, several lines of research point to this possibility. From early to late adolescence, emotion regulation shifts from amygdala-driven reactivity to prefrontal cortex-mediated top-down control (Nitin Gogtay et al., 2004), enabling older adolescents not only to dampen negative affect but also to cultivate positive low-arousal states (Silvers et al., 2012). Developmentally, mid-adolescents exhibit greater emotional granularity and self-awareness, allowing them to differentiate and harness nuanced positive emotions for sustained engagement (Gotlieb et al., 2024). Collectively, the evidence reviewed above points to the possibility of grade-level differences in these mediating pathways. However, there is currently a lack of empirical research examining these developmental differences, highlighting a need for further investigation.

### 2.4 The present study

The aforementioned reviews indicate that, while the influence of psychological resilience on learning burnout is well-established, the specific roles of the four categories of academic emotions as mediators remain unclear. It is uncertain which academic emotions (low- vs. high-arousal) exert significant mediating effects, and whether these effects vary across grades. This study aims to examine the mediating role of academic emotions and its grade-level differences.

Drawing on the Demand-Resources Model (DRM), Control-Value Theory (CVT), and the Broaden-and-Build Theory (Fredrickson, 2001; Pekerun et al., 2007; Bakker and Demerouti, 2014), we construct an integrated framework. From the DRM perspective, academic demands deplete self-regulatory resources, increasing burnout risk. Psychological resilience, as a personal resource, buffers this depletion and enhances control and self-efficacy. CVT further explains that resilience strengthens control-value appraisals, thereby reducing negative emotions and promoting positive ones. The Broaden-and-Build Theory suggests that positive emotions broaden cognitive repertoires, creating upward spirals, whereas negative emotions narrow attention and consume resources. Thus, resilience protects against burnout through three mechanisms: buffering resource depletion, enhancing control-value appraisals, and initiating positive emotional–cognitive cycles. Crucially, these three mechanisms are not independent or sequential. Rather, they are conceptually interwoven: buffering resource depletion enables more adaptive control-value appraisals, which in turn generate sustained low-arousal emotions, and these emotions—due to their chronic nature—feed back to conserve or replenish self-regulatory resources, shaping subsequent resilience efficacy, such as enjoyment and anxiety associated with learners‘self-efficacy which is one of control factors from control-value theory (Sun et al., 2026). Theoretically, this constitutes a recursive feedback loop that aligns with the dynamic spirit of all three frameworks. Empirically, however, the present cross-sectional study does not test this entire loop; instead, it focuses on testing the critical directional mediating pathways from resilience through academic emotions to burnout.

Notably, academic stress is predominantly chronic and diffuse, which tends to induce low-arousal emotions (e.g., boredom, helplessness, calmness). High-arousal emotions (e.g., excitement, anxiety) are typically transient and triggered by acute events, making them less stable as mediators. Therefore, we propose that low-arousal emotions—due to their enduring presence in daily academic life—will serve as consistent partial mediators, whereas high-arousal emotions will not. Research on adolescent brain development suggests grade-level differences. From early to late adolescence, there is a shift from amygdala-dominated reactivity to prefrontal cortex-mediated regulation (Gogtay et al., 2004; Gee et al., 2013). Thus, lower-grade students may rely more on regulating negative low-arousal emotions, while higher-grade students may additionally harness positive low-arousal emotions.

Based on this integrated theoretical perspective, the objective of this study is to test the following two hypotheses are proposed. Firstly, H_1(1): L_ow-arousal academic emotions will significantly mediate the relationship between psychological resilience and learning burnout, whereas high-arousal academic emotions will not demonstrate significant mediating effects. Secondly, H_1(2)_: The mediating effects of academic emotions will differ across grade levels, specifically: among higher-grade students, positive academic emotions (particularly positive low-arousal emotions) will demonstrate a stronger mediating effect; among lower-grade students, negative academic emotions (particularly negative low-arousal emotions) will exhibit a more prominent mediating effect.

This study is the first to examine both general and grade-specific mediation effects of low- vs. high-arousal academic emotions. It provides empirical evidence on how academic emotions mediate the resilience–burnout relationship and offers a theoretical foundation for grade-tailored interventions.

## 3 Research design

### 3.1 Ethics statement

With the approval of The Ethics Committee of the ****University (No. 20230005), a cluster sampling method was employed for practical feasibility and time efficiency. Following obtaining written consent from both the parents and the students, an anonymous online survey was conducted from December 1 to December 30, 2023.

### 3.2 Subjects

Students were able to complete the survey at home using their mobile phones or laptops during lunch breaks or after school in the evening. Participants were recruited from four schools in ****(2urban, 2rural, local society is characterized by a distinct urban–rural dual structure, therefore, conducting cluster sampling of classes from both urban and rural areas offers strong representative and feasibility for research purposes), with 45% from low-income families. A total of 885 questionnaires were distributed to middle school students across Grade One to Three. 83 questionnaires were excluded due to missing or inconsistent responses, and 61 questionnaires were not returned by the students. Consequently, 739 valid questionnaires were obtained, resulting in an effective response rate of 83%. The sample comprised 239 students from Grade One, 233 students from Grade Two, and 267 students from Grade Three, with a gender distribution of 370 males and 369 females. The average age of the participants was 14.3 years, with a standard error of 1.1.

According to the established guidelines (Bentiler and Chou, 1987; Loehlin, 2004; Bentiler and Chou, 1987; Loehlin, 2004; Boomsma, 1982), the overall sample of 739 cases substantially exceeds the minimum requirement of 200 cases recommended for models of moderate complexity. For the grade-level subgroups, the sample sizes (*n* = 239, 233, and 267 for Grades 7, 8, and 9, respectively) all exceed the recommended minimum of 200 cases (Boomsma, 1982; Loehlin, 2004), supporting the adequacy of the sample for multi-group SEM analyses.

### 3.3 Measurement tools

The Adolescent Learning Burnout Scale was developed by Wu et al. (2010) (Aguayo-Estremera et al., 2023). The scale has 16 items, including three dimensions, physical and mental exhaustion (4 items), academic alienation (5 items) and low sense of achievement (7 items), which are scored a five-point scale ranging from 1 (very inconsistent) to 5 (very consistent). The sum of the scores of all 16 items is the total learning burnout score, which reflects the overall situation of the subjects’ learning burnout. The higher the score is, the worse the burnout. In the current study, the Cronbach’s Alpha of the questionnaire was.893, and the Cronbach’s Alpha for physical and mental exhaustion, academic alienation and low sense of achievement were 0.766, 0.792 and 0.848, respectively. The relationships between them ranged from 0.490 to 0.616. Confirmatory factor analysis revealed that *χ*^2^/df = 3.88, CFI = 0.97, TLI = 0.96, RMSEA = 0.06, and NNFI = 0.96, indicating that the questionnaire has good reliability and validity.

The Adolescent Psychological Resilience Scale was developed in 2008 (Yueqin and Gan, 2008). The scale has 27 items, including two subscales, personal strength and social support. Personal strength includes three dimensions: goal focus, emotional control and positive cognition; social support includes family support and interpersonal assistance. The 5-point scoring method was adopted, ranging from 1 “totally inconsistent” to 5 “completely consistent”; the higher the total score is, the greater the level of psychological resilience. In the current study, Cranach’s Alpha of the total scale was. 868. Cronbach’s Alpha of personal strength and social support was 0.840 and 0.849, respectively, and the relationship between them was 0.90. Confirmatory factor analysis revealed that *χ*^2^/df = 3.29, CFI = 0.95, TLI = 0.95, RMSEA = 0.06, and NNFI = 0.93, indicating that the scale has good reliability and validity.

The Adolescent Academic Emotion Questionnaire was completed in 2007 (Yan and Yu, 2007). The questionnaire has 72 items and adopts a 5-point scoring method ranging from 1 (very inconsistent) to 5 (very consistent). The higher the score is, the more intense the emotional experience. The scale includes four dimensions: positive high-arousal of academic emotions, including 16 items related to pride, happiness and hope; positive low-arousal academic emotion, including 14 items related to satisfaction, calmness and relief; negative high-arousal academic emotion, including 17 items related to anxiety, shame and anger; and negative low-arousal academic emotion, including 25items related to boredom, helplessness, depression and fatigue. In the current study, the Cranach’s Alpha for positive high-arousal and low-arousal academic emotions is 0.826 and 0.873, the Cranach’s Alpha for negative high-arousal and low-arousal academic emotions is 0.870 and 0.890. An exploratory factor analysis was conducted on positive academic emotions and negative academic emotions, and it was found that depression belongs to negative high-arousal academic emotions. For this structure, Confirmatory factor analysis revealed that *χ*^2^/df = 3.32, CFI = 0.94, TLI = 0.93, RMSEA = 0.06, and NNFI = 0.91, indicating that the scale has good reliability and validity.

### 3.4 Statistical analysis

The statistical analysis proceeded in three phases, with each phase serving a distinct purpose in the analytic sequence. Phase 1: Common method bias test. Prior to hypothesis testing, common method bias was assessed using Harman’s single-factor test as a procedural check to determine whether the data were suitable for subsequent latent variable path analysis. Phase 2: Correlation analysis. Using SPSS 21.0, bivariate correlations among all study variables (psychological resilience, learning burnout, and the four types of academic emotions) were computed for the full sample and separately for each grade group. This analysis provided preliminary evidence for the relationships among variables, informing the specification of the subsequent mediation models. Phase 3: Hypothesis testing. Formal hypothesis testing was conducted using Mplus 8.3. Gender and parental education level were included as covariates in all SEM models. The hypothesis-testing procedure followed a three-step analytical strategy:

Step 1: Overall mediation model (testing *H_1_*). A parallel mediation model was estimated using the full sample The significance of indirect effects was assessed using bias-corrected bootstrapping with 5,000 resamples, with statistical significance determined by *p*-values derived from the bootstrap distribution. Step 2: Multi-group comparisons across grades (testing *H_2_*). To test whether the mediating mechanisms differ across grades, three sequential invariance tests were conducted: (a) configural invariance (model configuration), (b) measurement invariance (metric, scalar, and strict), and (c) structural invariance (structural weights, covariances, and residuals). Model fit comparisons were based on ΔCFI ≤ 0.010 and ΔRMSEA ≤ 0.015. Step 3: Grade-specific pairwise comparisons of path coefficients (further analysis of H_2_). To further identify which specific paths differed between grades, post-hoc pairwise comparisons of individual path coefficients were conducted using the Wald test (|*z*| > 1.96). Additionally, bias-corrected bootstrapping with 5,000 resamples was used to estimate grade-specific indirect effects within each grade subgroup.

## 4 Results

### 4.1 The common method bias test and correlation analysis

To assess the potential existence of deviation from the common method of questionnaire survey, the Harman single-factor test was employed through exploratory factor analysis. Without the application of rotation, 19 factors with eigenvalues greater than 1 were extracted. The first common factor accounted for 27.30% of the variance, which does not exceed the 40% threshold typically indicative of a significant interpretation rate. Furthermore, there was no notably high interpretation rate associated with any common factor. Consequently, it can be concluded that there is no substantial common method bias present in this study.

Learning burnout’s dimensions are strongly negatively correlated with dimensions of psychological resilience, the distribution of *r* values were from −0.66 to −0.47 (*p* < 0.01), moderately negatively correlated with positive academic emotions, the distribution of *r* values were from −0.73 to −0.11 (*p* < 0.01), and highly positively correlated with negative academic emotions (the distribution of *r* values were from 0.31 to 0.74) (*p* < 0.01) (The results about three grades shown in Tables 1, 2). Conversely, psychological resilience’s dimensions are negatively correlated with negative academic emotion (the distribution of *r* values were from −0.63 to −0.48) (*p* < 0.01), and positively correlated with positive academic emotion (the distribution of *r* values were from 0.29 to 0.67) (*p* < 0.01) (results about three grades shown in Table 1). These patterns preliminary justify the subsequent grade-general mediation analyses.

**Table 1:** The academic emotions, correlations with dimensions of resilience and learning burnout.

Variables	SS	PS	PHAE	PLAE	NHAE	NLAE	EBM	AA	LSA	SS	PS	PHAE	PLAE	NHAE	NLAE	EBM	AA	LSA
Three grades										Grade One								
SS	1	0.604	0.320	0.493	−0.473	−0.589	−0.526	−0.535	−0.502	1	0.530	0.348	0.431	−0.428	−0.558	−0.507	−0.469	−0.440
PS	0.604	1	0.484	0.664	−0.444	−0.649	−0.486	−0.593	−0.655	0.530	1	0.590	0.664	−0.396	−0.611	−0.545	−0.639	−0.654
EBM	−0.526	−0.486	−0.148	−0.395	0.520	0.638	1	0.569	0.335	−0.507	−0.545	−0.340	−0.461	0.489	0.689	1	0.568	0.420
AA	−0.535	−0.593	−0.361	−0.470	0.449	0.728	0.569	1	0.454	−0.469	−0.639	−0.535	−0.522	0.448	0.729	0.568	1	0.474
LSA	−0.502	−0.655	−0.450	−0.716	0.312	0.511	0.335	0.454	1	−0.440	−0.654	−0.570	−0.756	0.294	0.507	0.420	0.474	1
Grade two										Grade Three								
SS	1	0.614	0.296	0.511	−0.474	−0.579	−0.531	−0.539	−0.531	1	0.645	0.315	0.520	−0.496	−0.613	−0.522	−0.583	−0.522
PS	0.614	1	0.298	0.645	−0.507	−0.662	−0.515	−0.549	−0.635	0.645	1	0.586	0.675	−0.391	−0.653	−0.376	−0.610	−0.674
EBM	−0.531	−0.515	−0.078	−0.405	0.530	0.624	1	0.567	0.355	−0.522	−0.376	−0.039	−0.292	0.516	0.591	1	0.575	0.194
AA	−0.539	−0.549	−0.168	−0.423	0.470	0.714	0.567	1	0.445	−0.583	−0.610	−0.421	−0.503	0.423	0.749	0.575	1	0.475
LSA	−0.531	−0.635	−0.325	−0.689	0.371	0.517	0.355	0.445	1	−0.522	−0.674	−0.482	−0.686	0.233	0.497	0.194	0.475	1

PHAE, positive high-arousal academic emotion; PLAE, positive low-arousal academic emotion, HNAE, negative high-arousal academic emotion; LNAE, negative low-arousal academic emotion; EBM, Exhaustion of Body and Mind; AA, Academic Alienation; LSA, Low Sense of Achievement; PS, Personal Strength; SS, Social Support; LB, learning burnout only 0.078 and −0.039 are not significant, *p* > 0.01, the other correlations are significant, *p* < 0.01.

**Table 2:** The correlation among academic emotions, psychological resilience and learning burnout.

Variables	PR	PHAE	PLAE	NHAE	NLAE	PR	PHAE	PLAE	NHAE	NLAE
Three grades						Grade One				
PHAE	0.439	1				0.481	1			
PLAE	0.635	0.572	1			0.649	0.587	1		
NHAE	−0.513	−0.007	−0.370	1		−0.494	0.050	−0.317	1	
NLAE	−0.687	−0.242	−0.541	0.735	1	−0.695	−0.246	−0.516	0.743	1
LB	−0.751	−0.370	−0.626	0.549	0.789	−0.746	−0.354	−0.582	0.522	0.786
Grade Two						Grade Three				
PHAE	0.518	1				0.330	1			
PLAE	0.608	0.655	1			0.636	0.495	1		
NHAE	−0.472	−0.090	−0.367	1		−0.543	0.017	−0.396	1	
NLAE	−0.663	−0.344	−0.586	0.739	1	−0.686	−0.141*	−0.517	0.717	1
LB	−0.744	−0.568	−0.687	0.518	0.798	−0.752	−0.216	−0.603	0.581	0.778

−0.007, −0.050, 0.017, −0.090 are not significant, *p* > 0.05, the other correlations are significant, *p* < 0.05.

Further analysis of the correlations between variables within the three grades revealed that, as hypnosis, while the direction of the correlations between the variables remained consistent, the strength of the correlation between positive high-arousal emotions and learning burnout varied significantly across the three grades, with values of −0.354, −0.568, and −0.216, respectively (results about different grades shown in Table 2). These patterns preliminary justify the subsequent grade-specific mediation analyses.

### 4.2 The path analysis of resilience predicting burnout through mediating from emotions

**Figure 1:**
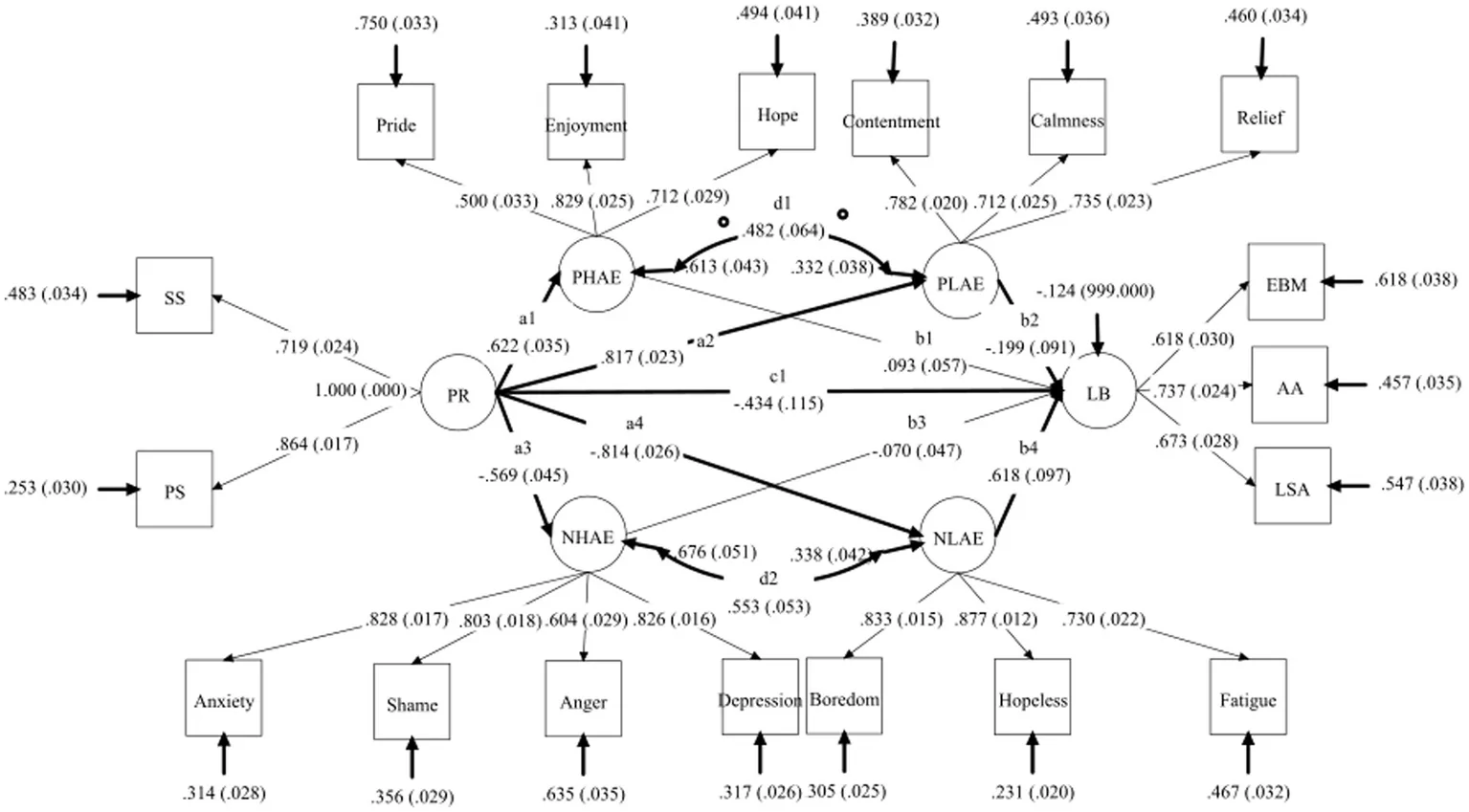
Resilience predicting burnout by mediators of academic emotions.

Structural equation analysis was performed on the path between psychological resilience mediating academic emotions and learning burnout. The estimation method was MLMV. The estimated parameters are shown in Figure 1. The indexes of the model are CMIN = 986.285, df = 124, *χ*^2^ = 7.95, CFI = 0.874, IFI = 0.844, RSME = 0.09, SRMR = 0.08. Although the model fit indices (CFI = 0.874, IFI = 0.844) did not meet the ideal standard of >0.90, they all exceeded the acceptable threshold of 0.85 (Hu and Bentler, 1999; Marsh et al., 2004) Additionally, both RMSEA (0.09) and SRMR (0.08) were below the critical value of 0.10, and the *χ*^2^/df ratio (7.95) also fell within an acceptable range due to its sensitivity to large sample sizes (Kline, 2023).

As for the total indirect effect, academic emotions explained 61.8% of the variance in learning burnout (*p* < 0.001), only low-arousal academic emotions have mediating effects on the relationship between psychological resilience and learning burnout. The mediating effects of negative and positive low-arousal academic emotions are significant, with effect values of −0.548 and −0.177 respectively, the two *p* < 0.05. The predictive effect of psychological resilience on low-arousal academic emotions is significantly higher than that on high-arousal academic emotions, and the predictive effect of low-arousal academic emotions on learning burnout is significantly higher than that on high-arousal academic emotions. a1 * b1 = 0.063, a3 * b3 = 0.044, they are different from the positive and negative values of the impact of psychological resilience on academic learning burnout, which means that psychological resilience has a certain masking effect on learning burnout through positive high-arousal and negative high-arousal, but this masking effect has not reached significance, and the *p* values are all greater than 0.05 (*p* = 0.117 and 0.142 > 0.05).

From the perspective of the path analysis, the predictive effect of psychological resilience on low-arousal academic emotions is significantly higher than on high-arousal academic emotions, and the predictive effect of low-arousal academic emotions on learning burnout is significantly higher than on high-arousal academic emotions (the results shown in Table 3). Therefore, although the overall effect of psychological resilience on academic burnout through the mediation of academic emotions is significant, only low-arousal academic emotions have mediating effects on the relationship between psychological resilience and learning burnout (shown in Table 3).

**Table 3:** The path parameters and mediating effects of the global model.

Comparisons between paths	EST	E.S	*z*	*p*		EST	E.S	*z*	*p*
a2-a1	0.329	0.056	5.873	0.00	a1 * b1	0.063	0.04	1.56	0.117
b2-b1	0.327	0.145	2.258	0.024	a2 * b2	−0.177	0.08	2.21	0.027
a4-a3	0.279	0.065	4.288	0.00	a3 * b3	0.044	0.03	1.46	0.142
b4-b3	0.585	0.109	5.378	0.00	a4 * b4	−0.548	0.091	6.02	0.000

### 4.3 The invariance of path structure between grades

Firstly, whether the path structure configure are consistent between the three grades was tested. Due to the limitation of the number of subjects in each grade (200 < *n* < 300), the MLMV estimation method was used to test the models of the three grades. All of the fitting indexes NFI, IFI, TLI, and CFI of the three grades were at the range from 0.82 to 0.87, RMSE was from 0.089 to 0.084, and all of *χ*^2^ were less than 5, *p* < 0.00.

**Figure 2:**
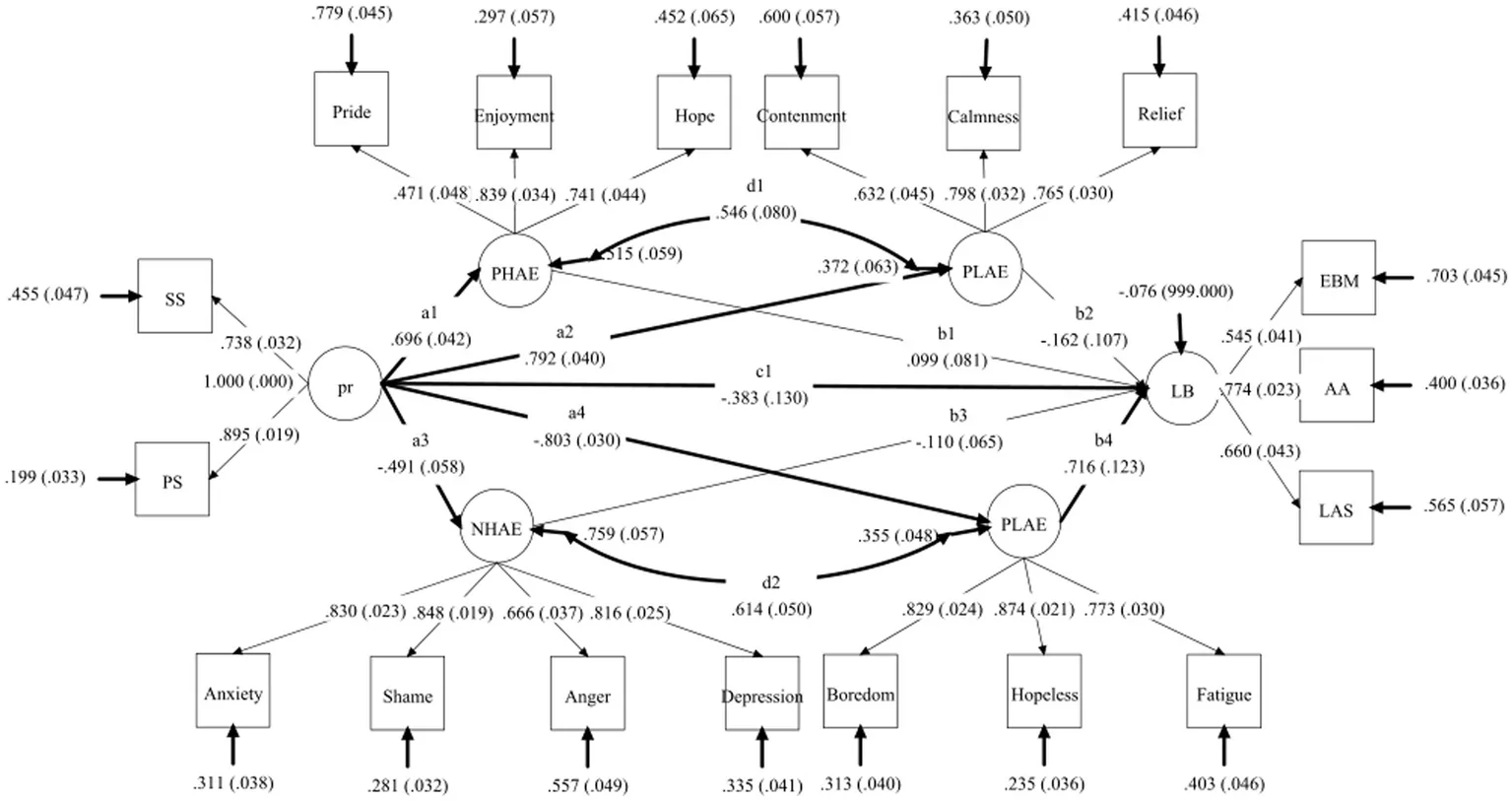
Resilience predicting burnout by mediators of academic emotions in Grade One.

**Figure 3:**
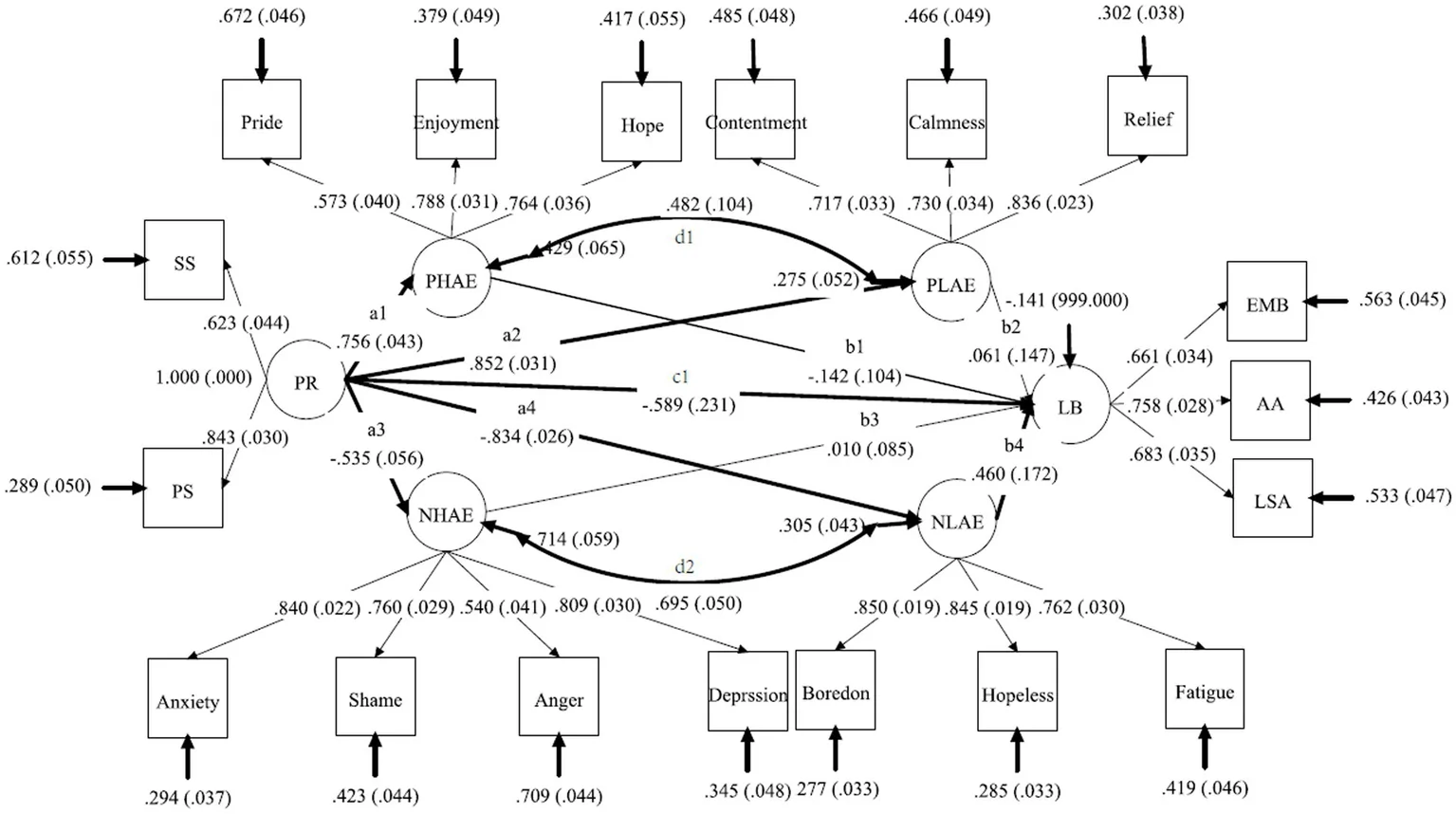
Resilience predicting burnout by mediators of academic emotions in Grade Two.

**Figure 4:**
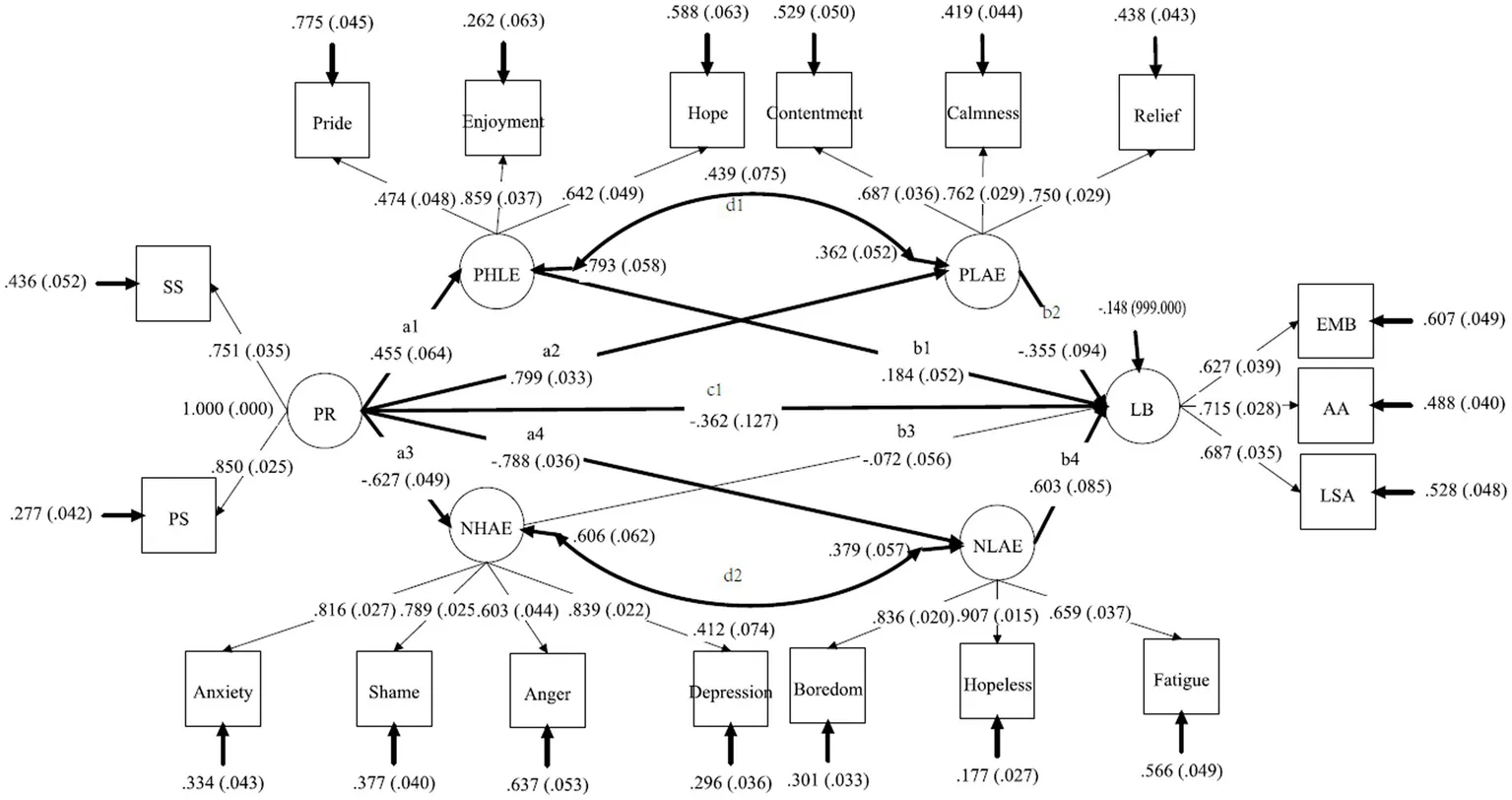
Resilience predicting burnout by mediators of academic emotions in Grade Three.

Although the comparative fit indices (CFI, TLI, NFI, IFI) fell below the ideal threshold of 0.90, they approached or exceeded the minimally acceptable level of 0.85 in several cases (Hu and Bentler, 1999), and the values of RMSEA (< 0.09) and *χ*^2^/df (<5) were within acceptable ranges (Schermelleh-Engel et al., 2003). More importantly, all indices showed remarkable consistency across grades. Therefore, despite less than optimal fit, the model was considered acceptable for further invariance testing given the following reasons: (a) the complexity of the model and the real-world educational data often lead to modest fit indices (Marsh et al., 2004); (b) the sample size for each group was limited, which can affect fit indices (Kenny and McCoach, 2003); (c) the primary focus was on comparing relative invariance across groups rather than achieving absolute fit (Chen, 2007). Additionally, the configural invariance model (without constraints) also demonstrated similar patterns: the fitting indexes NFI, IFI, TLI, and CFI are all between 0.82and 0.85, RMSEA is between 0.068 and 0.089, and *χ*^2^/df is all less than 5, *p* < 0.00, indicating that the configure of model is consistent between the three grades (Shown Table 4), they have the same configure between grades (shown Figures 1–4). The close similarity in fit across grades provided evidence that the configuration of the model was consistent, supporting its use for subsequent invariance testing. The differences in CFI between groups were within the recommended threshold of <0.01 (Cheung and Rensvold, 2002), further supporting configural invariance (Figures 1–4).

**Table 4:** The comparisons of models between grades.

Configural model between grades	CMIN	DF	*p*	NFI	IFI	TLI	CFI	RMSEA
Grade One	358.101	124	0.00	0.821	0.853	0.829	0.861	0.089
Grade Two	334.312	124	0.00	0.833	0.870	0.835	0.869	0.085
Grade Three	356.86	124	0.00	0.825	0.859	0.839	0.87	0.084
Unconstrained	1,638.56	372	0.00	0.827	0.861	0.826	0.859	0.068

Invariance between grades	CMIN	DF	*p*	ΔNFI	ΔIFI	ΔTLI	ΔCFI	ΔRSME
Measurement weights	32.65	24	0.112	0.005	0.005	−0.007	−0.003	−0.001
Measurement intercepts	97.23	36	0.00	0.01	0.011	−0.007	−0.006	−0.002
Measurement residuals	95.85	36	0	0.01	0.011	−0.005	−0.006	−0.001
Structural weights	45.36	18	0.00	0.005	0.005	−0.003	−0.003	0
Structural co-variances	0.626	2	0.731	0	0	−0.001	0	0.001
Structural residuals	19.19	14	0.158	0.002	0.002	−0.004	−0.001	0

Secondly, measurement invariance across grade levels was tested, with the results presented in Table 4. The chi-square difference test was statistically significant for the scalar (measurement intercepts) and strict (measurement residuals) invariance models (Δ*χ*^2^ = 97 and 95, *p* < 0.05) but not for the metric invariance (measurement weights) model (Δ*χ*^2^ = 32.65, *p* = 0.112 > 0.05). However, the changes in practical fit indices were minimal across all levels of invariance: all ΔCFI values were less than |–0.006| and all ΔRMSEA values were less than 0.002 These values meet the stringent criteria established by researchers (Cheung and Rensvold, 2002; Chen, 2007), which posit that a ΔCFI exceeding |–0.010| and a ΔRMSEA exceeding 0.015 indicate a meaningful deterioration in model fit. Therefore, metric invariance (equality of factor loadings) was supported. This confirms that the construct is measured equivalently across grades and provides a justified foundation for proceeding to test the invariance of the structural weights (path coefficients).

Lastly, the consistency of the path structure weight between grades was tested. ΔNFI, ΔNFI, ΔNFI, ΔNFI, ΔNFI, ΔNFI, ΔNFI, ΔNFI, ΔIFI, ΔTLI, and ΔCFI of the path structure weight were all greater than 0.003, Δ*χ*^2^ = 45.36, *p* < 0.00, indicating that the path structure differences between grades were significant. The consistency of structural covariance variation and structural variation explanation were tested, it was found that ΔNFI, ΔIFI, ΔTLI, and ΔCFI were alIFI, ΔTLI, and ΔCFI of the path structure weight were all greater than 0.003, Δ*χ*^2^ = 45.36, *p* < 0.00, indicating that the path structure differences between grades were significant. The consistency of structural covariance variation and structural variation explanation were tested, it was found that ΔNFI, ΔIFI, ΔTLI, and ΔCFI were alIFI, ΔTLI, and ΔCFI of the path structure weight were all greater than 0.003, Δ*χ*^2^ = 45.36, *p* < 0.00, indicating that the path structure differences between grades were significant. The consistency of structural covariance variation and structural variation explanation were tested, it was found that ΔNFI, ΔIFI, ΔTLI, and ΔCFI were alIFI, ΔTLI, and ΔCFI of the path structure weight were all greater than 0.003, Δ*χ*^2^ = 45.36, *p* < 0.00, indicating that the path structure differences between grades were significant. The consistency of structural covariance variation and structural variation explanation were tested, it was found that ΔNFI, ΔIFI, ΔTLI, and ΔCFI were alIFI, ΔTLI, and ΔCFI of the path structure weight were all greater than 0.003, Δ*χ*^2^ = 45.36, *p* < 0.00, indicating that the path structure differences between grades were significant. The consistency of structural covariance variation and structural variation explanation were tested, it was found that ΔNFI, ΔIFI, ΔTLI, and ΔCFI were alIFI, ΔTLI, and ΔCFI of the path structure weight were all greater than 0.003, Δ*χ*^2^ = 45.36, *p* < 0.00, indicating that the path structure differences between grades were significant. The consistency of structural covariance variation and structural variation explanation were tested, it was found that ΔNFI, ΔIFI, ΔTLI, and ΔCFI were alIFI, ΔTLI, and ΔCFI of the path structure weight were all greater than 0.003, Δ*χ*^2^ = 45.36, *p* < 0.00, indicating that the path structure differences between grades were significant. The consistency of structural covariance variation and structural variation explanation were tested, it was found that ΔNFI, ΔIFI, ΔTLI, and ΔCFI were alIFI, ΔTLI, and ΔCFI of the path structure weight were all greater than 0.003, Δ*χ*^2^ = 45.36, *p* < 0.00, indicating that the path structure differences between grades were significant. The consistency of structural covariance variation and structural variation explanation were tested, it was found that ΔNFI, ΔIFI, ΔTLI, and ΔCFI were all less than 0.003, and Δ*χ*^2^ were 0.626 and 19.19 respectively, *p* > 0.00. According to the fit criteria which has been cited above, this means that there are invariances of both structure covariance and residuals. But maybe there is variance of structure weights across grades (results shown in Table 4). This indicates that the predictive effects of psychological resilience on academic emotions, and of academic emotions on burnout, are not uniform. Specifically, the path coefficients a1, a2, a3, a4 (from resilience to emotions in Figures 2–4) and b1, b2, b3, b4 (from emotions to burnout in Figures 2–4) may be significant between-group differences. Therefore, the grade-specific mediating effects of emotions should be analyzed.

### 4.4 The grade-specific mediating effects of emotions between grades

Firstly, the path coefficients of among latent variables (Tables 5, 6) were compared between grades. From the result of resilience on academic emotions shown in Table 5, resilience can significantly predict the four categories of academic emotions across grades. There is no significant grade difference in the impact of psychological resilience on negative low-arousal academic emotions, a2(1)-a2(2) = 1.739, a2(2)-a2(3) = −0.946, a2(3)-a2(1) = 0.836 (a and b denote the paths from psychological resilience to academic emotions and from academic emotions to learning burnout, respectively. In notations like 2(1), the first digit indicates the path number and the second indicates the grade level, with all subsequent codes following this convention.). There is no difference in the impact of psychological resilience on and negative low-arousal academic emotions between three grades, a3(1)-a3(2) = 0.415, a3(1)-a3(3) = −1.459, a3(2)-a3(3) = −0.975, a4(1)-a4(2) = −0.344, and a4(1)-a4(3) = −0.29, a4(2)-a4(3) = 0.057. There is no significant grade difference in the impact of psychological resilience on learning burnout, c1(1)-c1(2) = −1.258, c1(2)-c1(3) = 0.776, c1(2)-c1(3) = −0.638. This means that although the resilience can predict academic emotion, but there are differences between grades.

**Table 5:** The parameters of structural paths from grades.

Path	Grade One				Grade Two				Grade Three			
	EST	E.S	*T*	*p*	EST	E.S	*T*	*p*	EST	E.S	*T*	*p*
b1	0.099	0.081	1.22	0.22	−0.142	0.104	−1.36	0.17	0.184	0.052	3.51	0.00
b2	−0.16	0.107	−1.51	0.129	0.061	0.147	0.41	0.68	−0.355	0.094	3.77	0.00
b3	−0.11	0.065	−1.68	0.09	0.010	0.085	0.118	0.91	0.072	0.056	1.284	0.20
b4	0.71	0.123	5.82	0.00	0.46	0.172	2.66	0.01	0.603	0.085	7.12	0.00
a1	0.696	0.042	16.56	0.00	0.756	0.043	17.48	0.00	0.455	0.064	7.13	0.00
a2	0.85	0.031	27.87	0.00	0.852	0.031	27.87	0.00	0.799	0.033	24.46	0.00
a3	−0.54	0.056	−9.62	0.00	−0.535	0.056	−9.62	0.00	−0.627	0.049	−12.74	0.00
a4	−0.83	0.026	−32.52	0.00	−0.83	0.026	−32.52	0.00	−0.788	0.036	−21.61	0.00
c1	−0.38	0.130	−2.94	0.003	−0.59	0.231	−2.54	0.011	−0.362	0.127	2.862	0.01

**Table 6:** The mediating effects of academic emotions in the model of grades.

Effects	Grade One					Grade Two					Grade Three				
	EST	E.S	*T*	*p*	%	EST	E.S	*T*	*p*	%	EST	E.S	*T*	*p*	%
a1 * b1	0.07	0.06	1.19	0.23	0.07	−0.14	0.11	−1.33	0.18	0.10	0.09	0.03	2.66	0.00	0.09
a2 * b2	−0.12	0.08	−1.50	0.13	0.13	0.07	0.17	0.41	0.68	0.05	−0.29	0.07	−3.75	0.00	0.29
a3 * b3	0.05	0.03	1.66	0.09	0.05	−0.01	0.06	−0.12	0.90	0.00	0.05	0.03	1.22	0.22	0.05
a4 * b4	−0.54	0.11	−5.10	0.00	0.59	−0.51	0.18	−0.28	0.005	0.37	−0.49	0.07	−6.51	0.00	0.48
Indirect	−0.54	0.12	4.62	0.00	0.59	−0.59	0.27	2.18	0.029	0.42	−0.66	0.11	5.76	0.00	0.64
Total	−0.91	0.08	10.42	0.00	1	−1.37	0.134	−10.21	0.00	1	1.03	0.10	10.26	0.00	1

Secondly, a cross-grade comparison was conducted on the predictive paths from academic emotions to burnout. As shown in Table 5, neither positive high-arousal nor positive low-arousal academic emotions were significant predictors of burnout for students in Grade One or Two. Specifically, there is no significant difference among the three grades in the impact of negative high-arousal academic emotions on learning burnout, b3(1)-b3(2) = 1.293, b3(3)-b3(1) = −0.342, b3(2)-b3(3) = −1.574. There is non-significant difference among the three grades in the impact of negative low-arousal academic emotions on learning burnout, b4(1)-b4(2) = −0.715, b4(1)-b4(3) = 0.136, b4(3)-b4(2) = 0.08. It shows that the impact of psychological resilience on positive high-arousal academic emotions is significantly higher in the first and second-grade students than the third-grade students, a1(3)-a1(1) = −2.658 < −1.96, a1(3)-a1(2) = −4.196 < −1.96, a1(1)-a1(2) = 1.792. The impact of positive high-arousal emotions on learning burnout is greater in third grade students than in first and second grade students, b1(1)-b1(2) = −2.129 < −1.96, b1(1)-b1(3) = −1.709, b1(2)-b1(3) = 3.616 > 1.96. The impact of positive low-arousal academic emotions on learning burnout is significantly higher in the third grade than in the first and second grade, b2(3)-b2(1) = −0.946, b2(1)-b2(2) = 1.428, b2(3)-b2(2) = −2.415 < −1.96. This means that academic emotions have different mediating effects between grades.

Regarding the mediating effects, academic emotions played a significant role across all grades. Although academic emotions served as significant partial mediators in all three groups (with indirect effects of *β* = −0.54, −0.59, and −0.66, all *p* < 0.001), the specific patterns varied. Notably, negative low-arousal academic emotions (e.g., boredom, hopelessness) demonstrated a significant mediating effect consistently across all grades (effects of *β* = −0.54, −0.51, and −0.49). In contrast, positive low-arousal academic emotions (e.g., contentment, relaxation) served as a significant mediator only in Grade Three (*β* = −0.29, *p* < 0.001). The other tested indirect pathways were not statistically significant (Table 6). This indicates that while the overall mediation by academic emotions is present, the specific emotional pathways that are important differ between grade levels.

## 5 Discussion

This study aimed to elucidate the mediating roles of low- versus high-arousal academic emotions in the relationship between psychological resilience and learning burnout among middle school students, and to examine whether these mediating mechanisms differ across grades. Our findings reveal that only low-arousal academic emotions (both positive and negative) serve as significant mediators, whereas high-arousal emotions show no significant indirect effects. Moreover, negative low-arousal emotions mediate this relationship consistently across all three grades, while positive low-arousal emotions function as a mediator only among third-grade students. These results provide novel insights into the emotion-specific pathways linking resilience to burnout and highlight developmental shifts in emotion regulation during adolescence.

### 5.1 Why only low-arousal emotions mediate: emotion dynamics and neuroendocrine mechanisms

Consistent with prior research (Rushton et al., 2015; Cheng et al., 2020), the present study found significant correlations among psychological resilience, academic emotions, and learning burnout. Psychological resilience not only directly predicted lower burnout but also showed strong associations with both positive and negative academic emotions. These patterns support the Demand-Resources Model (Bakker and Demerouti, 2014): resilience, as a personal resource, helps students cope with academic demands, thereby reducing emotional exhaustion and cynicism.

However, a central finding is that only low-arousal academic emotions (positive low-arousal: e.g., calmness, contentment; negative low-arousal: e.g., boredom, helplessness) significantly mediate the resilience-burnout relationship, whereas high-arousal emotions (e.g., excitement, anxiety, shame) do not show significant indirect effects. This supports Hypothesis 1(1) and challenges the conventional emphasis on high-arousal emotions in academic settings.

Two complementary explanations based on emotion dynamics and neuroendocrine mechanisms are proposed. First, high-arousal emotions are typically triggered by acute, salient events and activate a rapid, transient response of the hypothalamic–pituitary–adrenal (HPA) axis (Bayes et al., 2021). Their intensity decays quickly due to neural habituation and allostatic regulation. In contrast, chronic academic stress—the dominant form of pressure in middle school—sustains a low-grade, persistent activation of the HPA axis, giving rise to diffuse low-arousal emotions such as boredom, fatigue, and helplessness (or, when coping is effective, calmness and contentment). The time course of emotions (Verduyn et al., 2009) implies that high-arousal states are too ephemeral to form stable mediating pathways, whereas low-arousal emotions, due to their longer duration and higher frequency in daily academic life, exert a cumulative impact on learning burnout.

Second, from a cognitive perspective, high-arousal emotions may create an attentional blink or narrow the scope of attention (Gable and Harmon-Jones, 2010), which, although momentarily focusing resources on threat or reward, fails to support the sustained self-regulated learning needed to prevent burnout. Low-arousal positive emotions, by contrast, broaden cognitive resources and promote flexible problem-solving, thereby building enduring resilience (Fredrickson and Branigan, 2005). Our finding that positive low-arousal emotions had a smaller effect size than negative low-arousal emotions suggests that in the context of chronic stress, reducing negative low-arousal emotions may be more immediately impactful than enhancing positive low-arousal emotions—a pattern congruent with the negativity bias (Baumeister et al., 2001).

Notably, high-arousal emotions were not completely irrelevant. They showed significant correlations with low-arousal emotions, and although their direct indirect effects were non-significant, they may influence learning burnout indirectly through their association with low-arousal emotions—a form of masking effect that did not reach significance but warrants further investigation.

### 5.2 Grade-specific mediating effects: developmental shift from emotion regulation to emotion cultivation

The most novel contribution of this study is the discovery that the mediating role of positive low-arousal academic emotions emerges only in third-grade students, whereas negative low-arousal emotions are consistent mediators across all three grades. This pattern supports Hypothesis 2 and aligns with developmental neuroscience research on prefrontal cortex maturation. During early adolescence (grades 1 and 2), amygdala-driven reactivity to stress dominates, and top-down regulatory control from the dorsolateral prefrontal cortex (DLPFC) is still developing (Arnsten, 2009; Gee et al., 2013). Students at these stages are more vulnerable to negative emotions and rely heavily on external support to down-regulate distress. Consequently, psychological resilience reduces burnout primarily by mitigating negative low-arousal emotions. Positive low-arousal emotions, although present, may not yet be effectively harnessed as a protective resource because the neural circuitry for savoring and intentionally sustaining positive states (e.g., reward-system–prefrontal coupling) is still maturing (Zhou et al., 2022). By Grade Three (ages 14–15), the DLPFC and its connectivity with the limbic system and default mode network have advanced significantly (Gotlieb et al., 2024). This allows students not only to dampen negative affect but also to actively generate and maintain positive low-arousal emotions through cognitive reappraisal, goal internalization, and self-referential processing. Thus, resilience in third graders operates through both pathways: reducing negative low-arousal emotions and enhancing positive low-arousal emotions. This developmental shift from emotion regulation (mainly dampening negative) to emotion cultivation (actively building positive) has important implications for school-based interventions.

Moreover, the academic context differs across grades. First graders face transition stress; second graders experience relative stability; third graders confront high-stakes entrance examinations and uncertainty about future academic tracks. These contextual demands shape which emotional pathways are activated. For third graders, the prolonged pressure may paradoxically increase the adaptive value of positive low-arousal emotions as a buffer against burnout, whereas for younger students, reducing boredom and helplessness is more urgent.

## 6 Conclusions, implications and limitations

### 6.1 Main conclusions

This study provides empirical evidence that low-arousal academic emotions are the primary mediators linking psychological resilience to learning burnout, whereas high-arousal emotions do not exhibit significant indirect effects. Critically, the mediating role of positive low-arousal emotions is grade-specific: it emerges only in third-grade students, while negative low-arousal emotions mediate this relationship consistently across all three grades. Thus, psychological resilience reduces learning burnout through two distinct pathways: (a) a general pathway via dampening negative low-arousal emotions (e.g., boredom, helplessness), which operates throughout middle school; and (b) a developmental pathway via enhancing positive low-arousal emotions (e.g., calmness, contentment), which becomes functional only in later adolescence as prefrontal regulatory capacities mature.

### 6.2 Theoretical contributions

From a theoretical perspective, this study advances the Control-Value Theory (Pekrun, 2006) and Broaden-and-Build Theory (Fredrickson, 2001) by specifying that low-arousal—rather than high-arousal—emotions are the primary carriers of resilience effects in chronic academic stress contexts. It also integrates a developmental neuroscience perspective (Gee et al., 2013; Gotlieb et al., 2024) into educational psychology, showing that the emotion-regulation functions of resilience shift from mainly negativity-focused in early adolescence to positivity-cultivating in later adolescence. Furthermore, by introducing the concepts of emotion dynamics (Verduyn et al., 2009) and the negativity bias (Baumeister et al., 2001), the study offers a mechanistic explanation for why low-arousal emotions dominate the mediation process.

### 6.3 Practical implications

Interventions should be grade-specific. For grades 1–2, prioritize reducing negative low-arousal emotions (e.g., boredom, helplessness) via mindfulness, problem-solving training, and teacher scaffolding that emphasizes effort and improvement rather than peer comparison. These strategies help students develop a sense of mastery (Gao et al., 2025), thereby counteracting helplessness and breaking the resource-depletion cycle. For grade 3, additionally cultivate positive low-arousal emotions (e.g., calmness, contentment) through task-break scheduling, reflective journaling, and cognitive reappraisal. Supporting students’ sense of autonomy enables older adolescents to generate and sustain positive emotional states (Wang and Reynolds, 2024) At the school system level, create a low-arousal supportive climate (e.g., reduce excessive homework and ranking notifications). In collectivist cultures like China, include parental psychoeducation to recognize and respond to low-arousal negative emotions rather than focusing solely on test scores. Intervention design should also consider individual differences in students’ vulnerability to negative emotions, providing differentiated scaffolding such as AI assistance for those with lower confidence or greater helplessness (Wang et al., 2025a,b).

### 6.4 Limitations and future directions

Several limitations should be noted. First, Although stratified cluster sampling across urban and rural schools was employed to enhance representativeness, the sample was drawn from only four schools, which may limit generalizability to other regions. Second, model fit (CFI = 0.87) was acceptable but not ideal; future research may use ESEM to improve fit (Marsh et al., 2014). Third, the sample was limited to western China, where a collectivist cultural background prevails. In such contexts, academic achievement is often viewed as a family obligation, and students’ emotions are closely tied to parental expectations, peer comparison, and face-saving (Markus and Kitayama, 1991). This cultural context may amplify the role of negative low-arousal emotions, as chronic academic pressure is reinforced by external monitoring and social evaluation. Future cross-cultural studies are needed to test whether the grade-specific mediation pattern found here generalizes to individualist societies, where academic stress is more individualized and emotional expression is less constrained by familial expectations. Fourth, interpretations of grade differences as reflecting prefrontal maturation remain speculative without neurobiological measures (e.g., fMRI, EEG). Fifth, potential confounders (e.g., prior achievement, school resources) were not included. Finally, all cross-sectional measures were self-report; multi-informant or behavioral indicators would strengthen future studies.

## Data Availability

The datasets presented in this study can be found in online repositories. The names of the repository/repositories and accession number(s) can be found in the article/supplementary material.
